# NetworkAnalyst 3.0: a visual analytics platform for comprehensive gene expression profiling and meta-analysis

**DOI:** 10.1093/nar/gkz240

**Published:** 2019-04-01

**Authors:** Guangyan Zhou, Othman Soufan, Jessica Ewald, Robert E W Hancock, Niladri Basu, Jianguo Xia

**Affiliations:** 1 Institute of Parasitology, McGill University, Montreal, Quebec, Canada; 2 Department of Natural Resource Sciences, McGill University, Montreal, Quebec, Canada; 3 Department of Microbiology and Immunology, University of British Columbia, Vancouver, British Columbia, Canada; 4 Department of Animal Science, McGill University, Montreal, Quebec, Canada

## Abstract

The growing application of gene expression profiling demands powerful yet user-friendly bioinformatics tools to support systems-level data understanding. NetworkAnalyst was first released in 2014 to address the key need for interpreting gene expression data within the context of protein-protein interaction (PPI) networks. It was soon updated for gene expression meta-analysis with improved workflow and performance. Over the years, NetworkAnalyst has been continuously updated based on community feedback and technology progresses. Users can now perform gene expression profiling for 17 different species. In addition to generic PPI networks, users can now create cell-type or tissue specific PPI networks, gene regulatory networks, gene co-expression networks as well as networks for toxicogenomics and pharmacogenomics studies. The resulting networks can be customized and explored in 2D, 3D as well as Virtual Reality (VR) space. For meta-analysis, users can now visually compare multiple gene lists through interactive heatmaps, enrichment networks, Venn diagrams or chord diagrams. In addition, users have the option to create their own data analysis projects, which can be saved and resumed at a later time. These new features are released together as NetworkAnalyst 3.0, freely available at https://www.networkanalyst.ca.

## INTRODUCTION

The scientific community is in the midst of a boom of transcriptomics yet there are few accepted and standardized bioinformatics tools to organize, analyze, visualize and interpret the resulting big data. To deal with the challenges from such datasets, new-generation bioinformatics tools must be high performance (i.e. scalable for large data or user traffic), intuitive to use (i.e. to enable complex analytics via simple interface) and universally accessible (i.e. web/cloud-based). Here, we introduce NetworkAnalyst 3.0 as a powerful web-based visual analytics platform for comprehensive profiling, meta-analysis and systems-level interpretation of gene expression data. NetworkAnalyst was first released in 2014 centered on PPI network analysis and visualization (1). It was soon updated (version 2.0) in mid 2015, with a completely revamped user interface and enhanced workflow for statistical meta-analysis of multiple gene expression studies (2). Over the years, we have made continuous updates and feature enhancements based on community feedback. According to Google Analytics, the public server has performed >220 000 data analysis jobs submitted from >14 000 users worldwide over the past 12-month period.

The development of NetworkAnalyst, and subsequent updates, have been driven by the practical data analysis challenges facing researchers from a wide variety of different areas. Addressing these needs has required different levels of effort and expertise. At the basic level, we have expanded support from the initial five model organisms to currently 17 species covering mammals, birds, bacteria, plants and parasites. In addition, many researchers do not have access to high-end computational infrastructure, and thus we have developed and made available a public Galaxy server to support raw RNAseq processing for all the 17 species. At the intermediate level, we have spent significant efforts in curating high-quality, comprehensive molecular interaction data to allow users to create gene regulatory networks, tissue or cell-type specific networks as well as gene co-expression networks to enable more biologically meaningful analysis. For gene expression analysis, we have implemented an interactive volcano plot and added the widely-used gene set enrichment analysis (GSEA) method (3). At the advanced level, we have spent most of our efforts on developing and improving visual analytics methods to address several key challenges in biological big data analysis. To address the ‘hairball’ effect associated with large network visualization, we have implemented 3D and VR network visualization. For networks with hierarchical structures such as enrichment network, we have developed a ‘meta-node’ feature which can be expanded to show more details upon user click. To overcome the limitations of Venn diagrams and chord diagrams, we have developed simple yet powerful heatmaps to allow users to intuitively compare gene lists of varying sizes for meta-analysis. Finally, NetworkAnalyst now allows users to save their data analysis projects and resume analysis later. Meanwhile, we have performed thorough code refactoring, updated the framework, and enhanced the user interface to significantly improve its efficiency and user experience. We have also updated the frequently asked questions (FAQs) and have added new tutorials for first time users. All these changes and updates have been released as NetworkAnalyst 3.0. It is now available freely at https://www.networkanalyst.ca.

## PROGRAM DESCRIPTION AND METHODS

**Figure 1.**
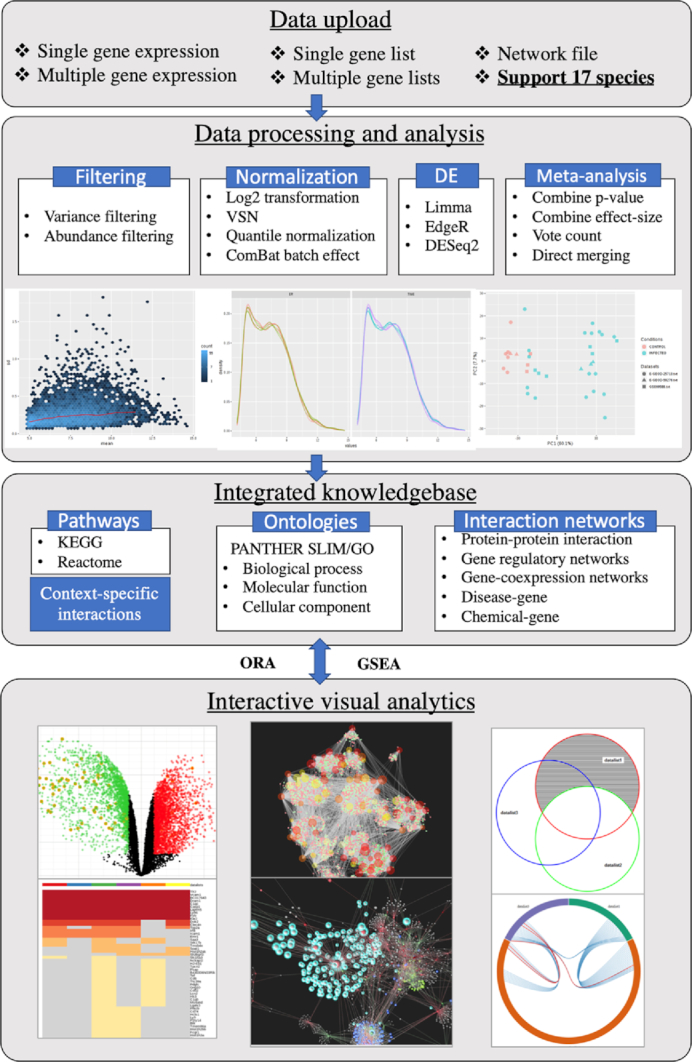
Overview of the workflow of NetworkAnalyst 3.0.

NetworkAnalyst accepts five types of data inputs - one or multiple gene lists, a single gene expression data table, multiple gene expression data tables, raw RNAseq reads as well as common network files. To start analysis, users can click the corresponding circular menu from the NetworkAnalyst home page. Each data input corresponds to a data analysis module with specific data processing steps. The analysis results will be presented in several highly interactive visual analytics methods with built-in support for functional enrichment analysis against multiple libraries available from our knowledgebase. The main workflow of NetworkAnalyst is summarized in Figure 1. In the following sections, we will focus primarily on the new or improved features introduced in the NetworkAnalyst 3.0. Other features can be found in our prior publications (1,2,4,5).

### Enhancing gene expression analysis

Given the prevalence of transcriptome studies across life sciences, we have spent substantial effort in improving both the capacity and the workflow for gene expression analysis, with a particular focus on RNAseq data analysis and interpretation.

#### Raw RNAseq processing

NetworkAnalyst now features a Galaxy-based pipeline for processing raw RNAseq, which includes trimming, quality checking, read mapping and quantification. In particular, we have implemented both the classical spliced aligner—HISAT2 (6), as well as the ultra-fast pseudoalignment based method—kallisto (7) to support raw RNAseq mapping for the 17 species. The resulting gene count tables can then be used for gene expression analysis as described below.

#### Gene expression profiling

To enable more refined data analysis and to improve the user experience, we have expanded the previous single-page gene expression analysis module into multiple pages spanning data upload, quality check, normalization and differential expression analysis steps. Both the quality check page and the normalization page include a number of diagnostic plots to provide different perspectives on the data. For instance, users can view the distributions of gene expression values across samples (box plots) or experimental factors (density plots), and the effects of different normalization methods on sample clustering can be visualized via PCA plots. All these figures can be downloaded as high-resolution images for publication. Differentially expressed genes (DEG) can be identified using limma (8), edgeR (9) or DESeq2 (10). Users can further select different parameters based on their study designs and comparisons of interest.

#### Interactive volcano plot

**Figure 2.**
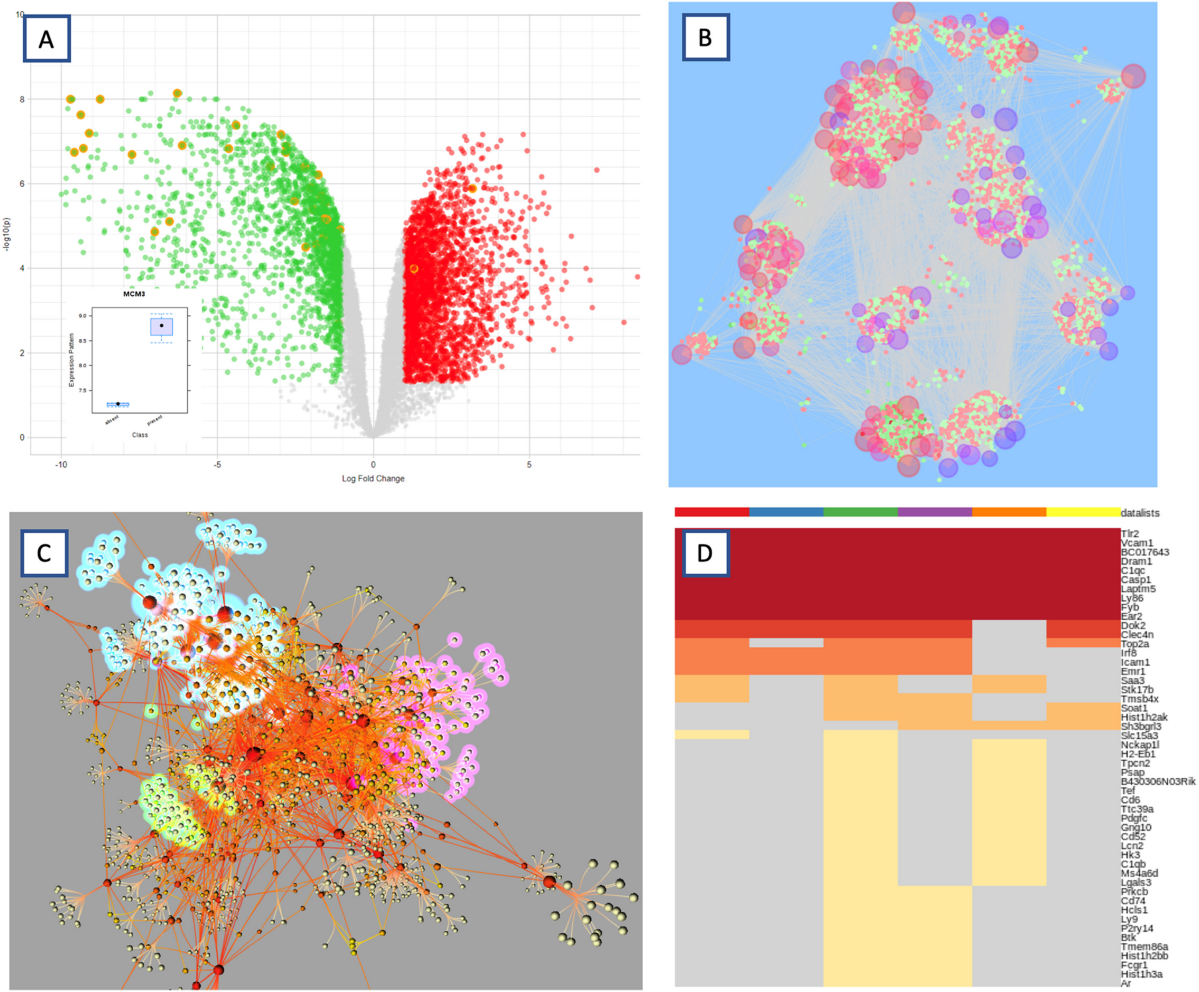
Screenshots of selected features introduced in NetworkAnalyst 3.0. (**A**) Interactive volcano plot. Users can click a data point to view the corresponding boxplot or click a function name to highlight the corresponding genes (shown in orange border). (**B**) Enrichment network with meta-nodes. Users can double click a meta-node (large semi-transparent circles) to view all its associated genes (small solid circles). (**C**) 3D network viewer displaying a force-directed tissue-specific PPI network with several modules highlighted; D) Multi-list heatmap viewer. Users can intuitively identify and select shared or unique gene subsets and then perform enrichment analysis.

This is a simple yet powerful visualization method that integrates statistical significance (p values) and biological significance (fold changes) to allow users to quickly identify the most promising gene candidates from differential expression analysis results. The interactive volcano plot was implemented based on the canvasDesigner package (11). Users can directly click any data point to view the corresponding gene name and its expression profile as a boxplot. Users can perform enrichment analysis on all DEG, up-regulated DEG, down-regulated DEG, as well as genes in the current selection. Double clicking any returned function name will highlight the corresponding genes in the volcano plot. A screenshot is shown in Figure 2A.

#### Gene Set Enrichment Analysis (GSEA)

In the previous versions of NetworkAnalyst, enrichment analysis was limited to over-representation analysis (ORA) on DEGs identified based on user selected cut-offs. Cut-off free methods, such as GSEA (3), utilizing the entire list of genes to compute functional enrichment, allows the detection of subtle yet consistent changes in gene expression profiles. GSEA in NetworkAnalyst is based on the high-performance *fgsea* R package (12). As GSEA requires a list of ranked genes as input, how to order the genes is an important parameter. NetworkAnalyst offers four robust gene ranking methods (moderated T-test, signal-to-noise ratio, fold change and statistics from the current DE method) based on a recent benchmark study (13). The results can be visualized as interactive heatmaps or enrichment networks. The heatmap visualization tool shows detailed gene expression patterns underlying individual functions; while the enrichment network tool (discussed further below) provides an overview of all enriched functions with similar ones connected by edges. A screenshot of an enrichment network is given in Figure 2B.

### Expanding molecular interaction knowledgebase

Biological networks provide an intuitive framework to help understand complex molecular interactions. While PPI networks are widely used to aid in the interpretation of gene expression data, it is clear that other types of networks are also needed to obtain deeper mechanistic insights. For example, gene regulatory networks incorporating transcription factors (TFs) or microRNAs (miRNAs) are critical to infer causal link of molecular interactions, while applying tissue or cell-type specific PPI can greatly reduce false positives. In addition, gene co-expression networks based on large-scale gene expression studies can complement networks based on experimental evidence to facilitate novel hypothesis generation (14). We have spent extensive efforts to expand the underlying molecular interaction knowledgebase as discussed below.

#### Context-specific networks

Our newly added human tissue-specific PPI data comes from the DifferentialNet database (15), covering 42 different tissues. The interaction data were generated by mapping tissue specific co-expression data from GTEx (16) to experimentally detected PPI data from four major interaction databases. The tissue-specific co-expression data comes from the TCSBN database (17), covering 46 tissues. The cell type specific co-expression data comes from Immuno-Navigator (18), covering 24 immune cell types in mouse and human.

#### Gene regulatory networks

Transcriptional and post-transcriptional gene regulation plays important roles in many biological processes and cellular functions. We have added two key players in gene regulation: TFs and miRNAs. TarBase (19) and miRTarbase (20) have been used to obtain experimentally validated miRNA–gene target information, while ENCODE (21), JASPAR (22) and CHEA (23) have been used to obtain TF–gene target information. We also included the TF–miRNA–gene coregulatory networks built by RegNetwork (24).

#### Other biological networks

To address growing needs in toxicogenomics and pharmacogenomics, NetworkAnalyst now also includes protein-chemical interactions from the Comparative Toxicogenomics Database (CTD) (25) and protein-drug interactions from DrugBank (26). The CTD is a comprehensive public database of toxicogenomic information manually curated from the literature, providing key information on the effects of environmental chemicals. DrugBank is a public database specialized in drug molecular information, mechanisms of action and drug-target information for >10 000 drugs. Additionally, we have included gene-disease association networks for humans from DisGeNet (27) which is a comprehensive database covering most of the known human disease-specific genotype–phenotype relationships.

### Addressing the ‘Hairball’ issue

As biological networks become increasingly large and complex, they often suffer from the well-known ‘hairball’ effect which greatly reduces their practical utilities and uptake. Two general approaches can be performed to overcome this issue: trimming the default network to retain only those significant nodes/edges; and developing better visualization methods to reduce edge and node occlusions. In NetworkAnalyst 3.0, we have implemented new functions employing both approaches.

#### Network customization

The default networks are created by searching for direct interaction partners in the molecular interaction knowledgebase. They are generally known as the first-order interaction networks. For very small networks, users can further expand the networks to create the second-order networks. When there are a large number of query genes (‘seeds’), it is reasonable to focus only on the interactions among those seeds (i.e. zero-order networks). However, many seeds could become orphan nodes when switching to zero-order networks. A ‘gentle’ approach is to extract, from the first-order network, a minimal subnetwork that maximally connects those seeds, a process known as Prize-collecting Steiner Forest (PCSF) algorithm. In NetworkAnalyst 3.0, we have added the support for efficient PCSF-based subnetwork extraction (28), as well as many other empirical trimming methods (available under ‘Network Tools’) based on shortest paths, node degree or betweenness values.

#### Network visualization in 3D and VR

Shifting from 2D to 3D can be a potential solution as it provides users a larger space for network layout and additional viewing angles. Although the hairball effect may still be problematic in 3D visualization, users will have more interaction freedom. Additionally, it may help expose some patterns otherwise undetectable in 2D visualization. Our implementation enables highly interactive network exploration in 3D space and allows extensive customization in terms of color, opacity, shading, etc. A screenshot of a 3D network generated by NetworkAnalyst is shown in Figure 2C. We have also added a virtual reality (VR) version of the 3D network based on the A-Frame framework (https://aframe.io/). VR brings to the table not only an immersive experience but also a much larger field of view that will not be limited by the size of the computer screen. Users with a compatible VR device (such as Oculus Rift) can view the network through web browsers. Please note our current implementation of the VR network is still in its prototype stage. We intend to develop a fully featured VR environment for 3D network visual exploration in the near future.

### Powering multi-list comparisons through visual analytics

NetworkAnalyst supports comprehensive meta-analysis of multiple gene expression tables through various statistical methods. In many cases, however, researchers may simply have a number of different lists of DEGs generated from different studies or different comparisons from the same studies, for which they wish to compare and analyze. This observation has been demonstrated by the tremendous popularities of several web-based tools dedicated to functional interpretation of a given gene list, such as WebGestalt (29), g:Profiler (30) and Enrichr (31). The research community is increasingly interested in comparing results across multiple studies, a key feature missing in the aforementioned tools is the ability to perform meta-analysis of multiple gene lists to identify their shared as well as unique functions. There is an unmet need for intuitive yet flexible bioinformatics tools to allow researchers to easily compare multiple gene lists to gain biological insights.

In NetworkAnalyst 3.0, multiple gene lists can be easily uploaded using the gene list module. Users simply insert a ‘//’ line to separate different gene lists when using the text area directly, or upload each gene list as an individual file. Please note NetworkAnalyst can also accept gene lists submitted programmatically as external requests based on our specified RESTful API (https://www.networkanalyst.ca/faces/docs/Resources.xhtml). After ID checking and conversion, users can visually compare different lists and perform enrichment analysis on a subset of genes generated from different set operations (i.e. unique, union, intersections for selected gene lists) using multiple visual analytics tools. A Venn diagram is probably the most straightforward way to compare a few gene lists - up to four gene lists are supported in our current implementation of Venn diagram. A chord diagram is also a popular visualization method to show pair-wise relationships between genes in multiple gene lists. However, a chord diagram can become too crowded when there are large number of genes and connections (>1000). To address these limitations, we have implemented two new methods to support the meta-analysis of genes and gene lists of arbitrary sizes.

#### Multi-list heatmaps

Heatmaps are a very popular visualization method for gene expression data. When used for visualizing multiple gene lists, heatmaps are able to show the presence or absence of genes in particular gene list in addition to the fold-change patterns. This form of presentation provides an overall picture of how the DEGs are shared across multiple lists. Our implementation allows users to directly click-and-drag to select a ‘patch’ of interest and perform enrichment analysis on the selected genes. Figure 2D shows a screenshot of the multi-list heatmaps in which different colors represent the frequencies of the genes appearing across all gene lists.

#### Enrichment network

To improve the interpretability of the results from enrichment analysis, we have implemented an interactive enrichment network viewer based on a similar concept introduced by ClueGO and EnrichmentMap (32,33). Users can now visualize the relationships among enriched function terms and their associated genes in a similarity network (Figure 2B). By default, the viewer shows a global enrichment network in which nodes represent functions and edges are determined by the overlap ratio between genes associated with the two functions. These nodes are implemented as meta-nodes. Users can double click to expand any meta-node to view its associated genes. Our implementation allows users to easily customize the style of the network (colors, layout, etc.) or to extract a subnetwork based on selected functions of interest.

### Enabling resumable and reproducible data analysis

There is a growing interest in the bioinformatics research community to develop solutions for sharing data and analysis steps to support publications and scientific claims (34,35). Due to the wide array of visual analytics methods available in NetworkAnalyst, users may not be able to complete their analyses in a single session. This can partially explain the >220 000 data analysis jobs submitted to NetworkAnalyst from ∼14 000 users over the past year—it is likely that many jobs were re-analyzing the same datasets submitted from the same users. There is a need for NetworkAnalyst to store user data and analysis steps to allow users to resume their data analysis later.

In NetworkAnalyst 3.0, we have developed a project management component as an initiative to address the challenges associated with reproducible research. Users can now create up to 10 projects. These projects can be loaded, updated or deleted. Within each project, all key analysis steps are tracked. Since these projects need to be stored securely, users need to create an account to manage their projects. It should be noted, however, that creating an account, is not required for using any data analysis module in NetworkAnalyst 3.0. 

### Implementation

NetworkAnalyst 3.0 was implemented based on the PrimeFaces (v6.2) component library (http://primefaces.org/) and R (version 3.5.1). The various visual analytics methods have been developed based on several powerful JavaScript libraries including sigma.js (http://sigmajs.org) for 2D interactive network visualization, three.js (https://threejs.org) for 3D network visualization and canvasXpress (https://canvasxpress.org) for heatmaps and volcano plots. The system is hosted on a Google Cloud *n1-highmem-8* instance (52GB RAM and eight virtual CPUs with 2.6 GHz each). The project management component has been developed as a microservice hosted on a separate server using Spring Boot and Spring Security. As a web-based tool, NetworkAnalyst is mainly designed to support analysis of gene expression data generated from small to medium-sized studies. For raw RNAseq data processing, our Galaxy Server offers 100 GB disc space per user by default (∼30–50 samples dependent on the organisms and sequencing depth); For gene expression table, users can upload files with a maximum size of 50 MB (∼200 samples with ∼25 000 genes for each sample). For meta-analysis, users can upload up to 1000 samples in total. For large-scale studies, we recommend users to first process their data locally and upload gene lists for network analysis and visual exploration.

### Comparison with other web-based tools

Table 1 shows the comparisons between NetworkAnalyst 3.0 and several other well-known web-based tools dedicated to functional profiling of transcriptomics data, including WebGestalt (29), g:Profiler (30) and Enrichr (31). The WebGestalt web application, first released in 2005, provides comprehensive enrichment analysis for 12 selected organisms and also supports user-supplied functional enrichment categories. The g:Profiler tool suite, first released in 2007, provides the broadest species coverage by supporting >200 species and corresponding gene ID conversions. Additional features include mapping human single nucleotide polymorphism (SNP) to gene name as well as ortholog search. The Enrichr web server, first released in 2013, provides the broadest functional coverage by supporting enrichment analysis against >100 gene set libraries. A key contribution of Enrichr is its curation effort, and allowing users to download their curated gene sets. Another unique feature of Enrichr is its support for BED file as input for enrichment analysis. These three tools are powerful web-based platforms that offer rich annotations for a given gene list. In contrast, NetworkAnalyst distinguishes itself from other web-based tool by providing cutting-edge network visualization, versatile visual analytics, comprehensive support for gene expression profiling, meta-analysis and multi-list comparisons. NetworkAnalyst 3.0 offers an end-to-end solution for RNAseq analysis - from raw reads mapping to differential expression analysis and identification of important pathways and functions.

**Table 1.** Comparison of NetworkAnalyst with other web-based tools. Symbols used for feature evaluations with ‘√’ for present, ‘-’ for absent, and ‘+’ for a more quantitative assessment (more ‘+’ indicate better support). The URLs for each tool are given below.

Tools	NetworkAnalyst	WebGestalt	g:Profiler	Enrichr
Inputs	Gene lists, gene expression data, network files	Gene lists	Gene lists	Gene lists, BED file
Organisms	17 species	12 species	213 species	6 species
**Gene expression analysis**				
RNAseq processing	√	-	-	-
DE analysis	√	-	-	-
Enrichment analysis	++	+++	++	+++
Knowledgebase	++	+++	+++	+++
**Network construction and visualization**				
Network types	+++	++	+	+
3D/VR network	√	-	-	-
**Meta-analysis and visual analytics**				
Multiple lists	Enrichment analysis on any sets (union, intersection, unique)	Enrichment analysis on individual lists		-
Multiple tables	√	-	-	-
Heatmap view	√	√	√	√
Chord diagram	√	-	-	-
Venn diagram	√	-	-	-
Enrichment network	√	√	-	√
Volcano plot	√(genes)	√ (gene sets)	-	-

• NetworkAnalyst: https://networkanalyst.ca/

• g:Profiler: https://biit.cs.ut.ee/gprofiler/

• WebGestalt: http://www.webgestalt.org/

• Enrichr: http://amp.pharm.mssm.edu/Enrichr

## CONCLUSIONS

NetworkAnalyst 3.0 is a unique online visual analytics platform specialized in transcriptome profiling, network analysis, and meta-analysis for gene expression data. NetworkAnalyst has been developed to address three unique gaps in the current landscape of bioinformatics tools. Firstly, NetworkAnalyst aims to provide a web-based tool for creating and visualizing biological networks to complement the widely used stand-alone tools such as Cytoscape (36). We will continue to add new features with a special consideration of emergent revolutions in web technologies (i.e. cloud, WebVR and browser computing) in the coming years. Secondly, NetworkAnalyst has filled a unique gap by enabling web-based meta-analysis of gene expression data. Gene expression meta-analysis is a very complex process and is usually performed by statisticians using R and Bioconductor packages rather than by average life science researchers. Curating as well as uploading and processing multiple datasets using online tools can be a challenging, unreliable (i.e. unstable connections) and time-consuming task. The implementation of the project management component is a first step towards addressing these concerns. Users can now save their projects (including datasets and steps) and resume analysis at a later time. In addition, the new multi-list comparison feature enables more flexible meta-analysis by accepting gene lists generated using users’ own favorite tools and methods. Finally, it is now widely accepted that the over-reliance on p-values (among other statistical missteps) have contributed to the current crisis in reproducible research (37,38). With the proliferation of datasets that are increasingly large and complex, there is a great need to design and develop novel and intuitive bioinformatics tools to better educate, empower and engage users. We believe that integrating a range of visual analytics tools together with ‘conversational’ data analysis steps as used by NetworkAnalyst is a promising approach towards addressing this issue.

## DATA AVAILABILITY

NetworkAnalyst 3.0 is freely available at https://www.networkanalyst.ca.
